# Penile cancer treatment costs in England

**DOI:** 10.1186/s12889-015-2669-2

**Published:** 2015-12-29

**Authors:** Sam T. Keeping, Michael J. Tempest, Stephanie J. Stephens, Stuart M. Carroll, Vijay K Sangar

**Affiliations:** Sanofi Pasteur MSD, Mallards Reach, Bridge Avenue, Maidenhead, Berks, SL6 1QP UK; Pharmerit Ltd, Enterprise House, Innovation Way, York, YO10 5NQ UK; The Christie NHS Foundation Trust, University Hospital of South Manchester, Wilmslow Road, Manchester, M20 4BX UK

**Keywords:** Burden of illness, Reimbursement, Penile cancer, England

## Abstract

**Background:**

Penile cancer is a rare malignancy in Western countries, with an incidence rate of around 1 per 100,000. Due to its rarity, most treatment recommendations are based on small trials and case series reports. Furthermore, data on the resource implications are scarce. The objective of this study was to estimate the annual economic burden of treating penile cancer in England between 2006 and 2011 and the cost of treating a single case based on a modified version of the European Association of Urology penile cancer treatment guidelines.

**Methods:**

A retrospective (non-comparative) case series was performed using data extracted from Hospital Episode Statistics. Patient admission data for invasive penile cancer or carcinoma in situ of the penis was extracted by ICD-10 code and matched to data from the 2010/11 National Tariff to calculate the mean number of patients and associated annual cost. A mathematical model was simultaneously developed to estimate mean treatment costs per patient based on interventions and their associated outcomes, advised under a modified version of the European Association of Urologists Treatment Guidelines.

**Results:**

Approximately 640 patients per year received some form of inpatient care between 2006 and 2011, amounting to an average of 1,292 spells of care; with an average of 48 patients being treated in an outpatient setting. Mean annual costs per invasive penile cancer inpatient and outpatient were £3,737 and £1,051 respectively, with total mean annual costs amounting to £2,442,020 (excluding high cost drugs). The mean cost per case, including follow-up, was estimated to be £7,421 to £8,063. Results were sensitive to the setting in which care was delivered.

**Conclusions:**

The treatment of penile cancer consumes similar levels of resource to other urological cancers. This should be factored in to decisions concerning new treatment modalities as well as choices around resource allocation in specialist treatment centres and the value of preventative measures.

**Electronic supplementary material:**

The online version of this article (doi:10.1186/s12889-015-2669-2) contains supplementary material, which is available to authorized users.

## Background

Invasive penile cancer is a rare neoplasm accounting for less than 0.5 % of all cancers worldwide [1] and most commonly affects men aged 50-70 years [2]. A recent epidemiological study reported an age-standardized incidence rate of 1.33 per 100,000 in 2009, a 21 % increase from estimates 30 years prior [3]. The majority of patients will have five year disease specific survival of over 90 % despite local recurrence being relatively common (~20 %) [4]. Associated risk factors include a lack of neonatal circumcision, phimosis, cigarette smoking, first sexual intercourse at an early age and human papillomavirus (HPV) infection [5, 6].

Despite a number of associated risk factors, the exact aetiology of penile cancer remains ambiguous. An estimated 60-100 % of penile intraepithelial neoplasia and 40-50 % of invasive penile tumours are however postulated to be attributable to HPV infection [5]. A review of 31 studies investigating the prevalence of HPV in invasive penile tumours found that 60.2 % of subjects had positive traces of HPV 16, followed by HPV 18 and HPV 6/11 (13.3 % and 8.13 %, respectively) [7].

Treatment of penile cancer is influenced by several factors, including tumour size and its location with respect to the glans. Surgical intervention is deemed the gold standard for high grade and high stage disease; ablative surgery exhibits lower risk of recurrence than conservative surgery (5 % vs. 27 %). The pathological assessment of surgical margins is imperative as positive results inevitably lead to local recurrence [8].

All treatments have a potentially detrimental impact on quality of life including issues due to disfigurement, sexual function, social interactions, self-image and self-esteem. Rehabilitation and post-treatment care are vital components in the treatment pathway for penile cancers and are often overlooked in cost-of-illness studies [9].

In a recent study conducted in France, penile cancer was associated with a total annual cost of €5.6 million (excluding expensive drug costs) based on 678 hospitalised patients. The total cost comprised €2.6 million hospitalisation costs, €2.3 million outpatient costs and €0.7 million daily allowance costs [6].

To our knowledge, no detailed estimates of the economic burden associated with penile cancer in England are currently available. Such data would be valuable for evaluating both the value of new treatments for penile cancer and also preventative strategies such as HPV vaccination programmes.

This study aimed to address this by examining both the total annual and per patient costs of treating penile cancer in England and also the total cost of treating a single case from diagnosis to discharge or death.

## Methods

The study was split into two phases. Firstly, to calculate annual patient numbers, along with mean annual total and per patient costs, a retrospective analysis was performed using data extracted from Hospital Episode Statistics (HES). Each hospital Finished Consultant Episode (FCE) results in the production of a summary of care discharge report, containing patient demographics, method of admission and discharge, the nature of treatment carried out during the stay including any comorbidities and complications, and the main diagnosis that preceded the hospital admission. Diagnoses are coded using the International Classification of Diseases, 10^th^ revision (ICD-10) either as primary, related, or significantly associated diagnoses.

Inpatient and outpatient records associated with any of the ICD-10 codes for penile cancer were extracted for the years 2006/07 to 2010/11 (2010/11 was representative of nine months provisional data). Since a hospital admission may include other ICD-10 code diagnoses where penile cancer is the secondary or tertiary diagnosis, a clinical expert verified that such instances should be included within the analyses to capture the broader burden of disease. ICD-10 codes for outpatient attendances were extracted for primary and secondary diagnoses only, as attendances are generally more disease specific in post-treatment care. The following ICD-10 codes were used to identify eligible records: C600 - Malignant neoplasm of prepuce, C601 - Malignant neoplasm of glans penis, C602 - Malignant neoplasm of body of penis, C608 - Malignant neoplasm, overlapping lesion of penis, C609 - Malignant neoplasm of penis, unspecified. Penile dysplasia was not included within this analysis as no specific ICD-10 code was available.

Approval for use of the HES data was provided by the information asset owner from the Health and Social Care Information Centre. The individual HES records extracted contained no sensitive data and were pseudonymised preventing the true identification of patients; analyses pertaining to HES records adhered to published regulations [10]. Ethical approval was not required as secondary analysis of HES data can be used to identify public health issues and for general medical research under existing protocol; the Health Research Authority decision tool corroborated this fact stating no ethical approval was required for this research [11].

Payment by Results is the payment framework for which healthcare providers are reimbursed by commissioners in England [12]. Nationally determined currencies and tariffs comprise the fundamental features of Payment by Results. The currency for inpatient care are Healthcare Resource Groups (HRG) which are setting-independent, clinically specified groups of diagnoses and interventions that consume similar levels of National Health Service resources. In contrast, the currency for outpatient care is based on treatment function codes.

Under Payment by Results, inpatient reimbursement is provided for a spell of care, which can potentially include multiple FCEs captured by HES. A spell is a more robust activity measure than an FCE because FCEs can easily be influenced (e.g. by transferring patients between consultants) in ways that spells cannot. To group episodes of care into spells, the 2010/11 local payment grouper was used [13]. A single core HRG was derived reflective of the nature of treatment given during a hospital stay.

Costs were considered from the healthcare payer perspective although ambulatory care, primary care, and costs pertaining to high cost drugs were not considered in the main analysis due to exclusion from HES. Hospital costs were calculated using the National Tariff 2010/11 [14] and Reference Costs 2006-10 [15] for HRGs excluded under the National Tariff. Costs were inflated using the consumer price index. Costs are presented (£, 2011 prices) as mean annual costs per patient and total costs per year (based on all inpatients and outpatients from 2006/07 to 2010/11). To compensate for the nine months provisional data in the final year, all costs, patient numbers and annual spells were multiplied by a correction factor (assuming no treatment seasonality) prior to mean and standard deviation calculations.

Under HRG4, some significant elements of cost and activity have been ‘unbundled’ from the core HRGs. The ‘unbundled component’ becomes a HRG in its own right as an addition to a core HRG. A spell of care may involve several procedures and care phases (for example diagnostic imaging, surgery and rehabilitation) each with an individual cost. Unbundling of the tariff separates the payment for that particular treatment, allowing different providers to receive the costs associated with different aspects of a procedure. Despite being constituents of unbundled HRGs, the procurement and delivery of chemotherapy and radiotherapy were disaggregated prior to analysis. Statistical analysis of the HES data was conducted using Statistical Analysis System Enterprise guide 4.3, and a costing algorithm was developed using Microsoft Excel 2007 with Visual Basic for Applications.

Due to limitations of the HES database which include the inability to distinguish cancer stage and type (initial versus recurrent; incident versus prevalent), the exclusion of treatment costs and the short data period in comparison to survival rates, a mathematical model of the treatment pathway was developed in order to estimate total per patient treatment costs. The treatment pathway was based on the European Association of Urology Guidelines on Penile Cancer [16], with probabilities for different treatment approaches taken from published local sources and expert opinion where necessary.

**Fig. 1:**
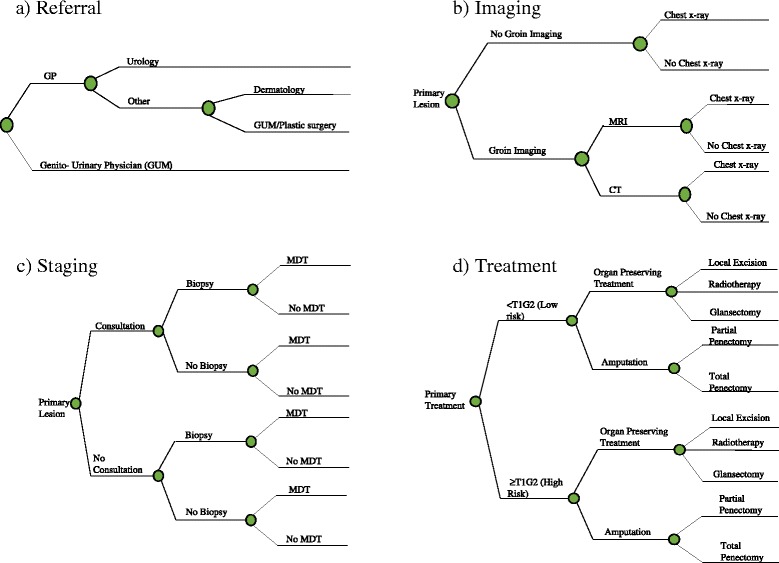
Decision tree pathways of referral, imaging, staging and treatment of primary tumour. CT; Computerised Axial Tomography; GP, General Practitioner; GUM, Genito-Urinary Medicine; MDT, Multi-Disciplinary Team; MRI, Magnetic Resonance Imaging Rich Text Editor, my Text Field 1 Editor toolbars

Firstly, a series of decision trees were constructed to estimate the costs of referral, staging and treatment for the primary tumour and local lymph nodes (Fig. 1). Each branch of the decision tree is associated with a probability based on publically available information and expert opinion (please see Additional file 1). A Markov extension to the model was then used to estimate the costs of follow-up, taking into account mortality and the probability of local, regional or distant relapse.

**Fig. 2:**
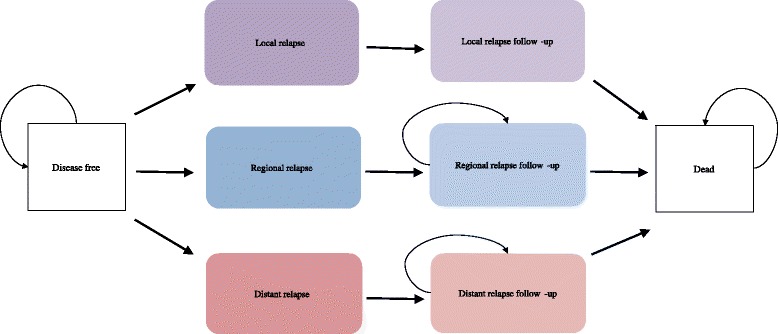
Structure of the Markov Model. Note: Patients were assumed to spend only one month in any of the three relapse states before either moving into the respective follow-up state or dying

Figure 2 shows the structure of the Markov model. Given that the risk of relapse tends to decrease over time, local and regional relapse probabilities were assumed to follow a Weibull distribution, with the probability of the latter falling to zero after five years [4]. For distant relapse, the risk was assumed to be constant up to two years after which it was assumed to be zero. The risk of death from any of the states in the model were also assumed to be constant.

**Fig. 3:**
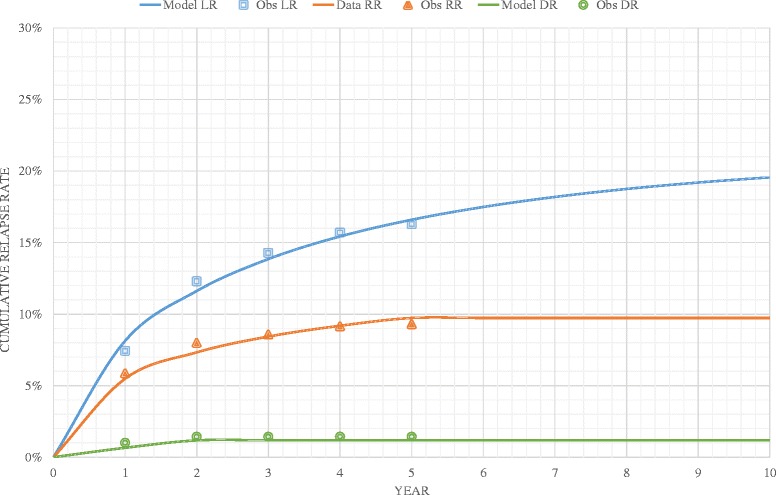
Observed versus predicted cumulative relapse rates. Obs, observed; LR, local relapse; RR, regional relapse; DR, distant relapse

Values for all parameters detailed above, including the time dependent risks for local and regional relapse, were estimated by fitting the model to published data from a large study of outcomes in penile cancer patients across two specialist treatment centres, one in Sweden and one in the Netherlands [4]. This was achieved using a recursive algorithm, with goodness-of-fit assessed using ordinary least squares. Figure 3 shows the fit of the calibrated model to data on the various types of relapse.

Consistent with the approach used in the HES analysis, costs applied in the model were taken from the 2010/11 National Tariff where the HRG associated with a treatment or intervention was known and included within the tariff provisions. The Reference Costs for the same year were used for all off-tariff payments. Four core scenarios were examined: no price inflation and no correction for regional market forces, no price inflation and correction for regional market forces, price inflation (assumed to remain constant at 2011/12 levels [17]) and no correction for regional market forces, price inflation and correction for regional market forces. In addition, a one way sensitivity analysis was carried out on the following inputs: mode of admission, costs of treatment, intensity of follow-up, stage at presentation, approach to primary tumour management and approach to lymph node management. Full details of the model inputs are available in Additional file 1.

## Results

Between 2006 and 2011, an average of 640 patients were admitted to hospital annually in England for invasive penile cancer; 48 patients attended outpatient facilities (Table 1). The mean age at presentation was 66 (data not shown).

**Table 1:** Mean annual number of patients, inpatient spells, outpatient attendances and cost per patient

	Inpatient			Outpatient		
	Number of spells (SD)	Number of patients (SD)	Cost per patient (SD)	Number of attendances (SD)	Number of patients (SD)	Cost per patient (SD)
Invasive Penile Cancer	1292 (56)	640 (39)	£3,737 (£145)	290 (156)	48 (13)	£1,051 (£604)
Carcinoma in Situ of the Penis	178 (28)	144 (21)	£1,316 (£82)	*	*	*

SD, Standard Deviation

In this table, *corresponds to a figure between 1 and 5. These have been suppressed as per the Hospital Episode Statistics analysis guide

On average, there were 1,292 hospital spells and 290 outpatient attendances each year (Table 1). The mean number of hospital spells and outpatient attendances per patient were 2 and 6, respectively. Mean length of stay was 5 days. Eighty-three per cent of hospital spells were elective admissions and 21 % were day case admissions. Excess bed days were observed in 17 % of elective and 4 % of non-elective hospital spells.

The most frequent inpatient HRGs were penile conditions with procedures of varying severity, representing 66 % of hospital spells (22 % major/intermediate procedures and 44 % minor procedures). Bladder and urinary tract disorders and procedures accounted for 7 % of hospital spells.

Minor outpatient procedures were observed in 6 % of all attendances, 25 % of which were related to pain. Follow-up consultations were the most frequent HRG observed in 84 % of attendances; first consultations accounted for 10 % of attendances. The most frequent treatment function code observed was clinical oncology (71 % of attendances).

Chemotherapy sessions were observed in 6 % of inpatient spells and 7 % of outpatient attendances. Radiotherapy sessions were observed in 1 % and 37 % of inpatient and outpatient attendances, respectively. Across settings, palliative care was associated with 1 % and rehabilitation with <1 % of hospital spells.

The mean annual costs per patient were estimated at £3,737 and £1,051, for inpatients and outpatients respectively (Table 1). For inpatients, £2,314,152 of the total cost was attributable to bundled costs which include all care received in a hospital setting excluding unbundled HRGs. Unbundled costs (excluding chemotherapy and radiotherapy) were equal to £34,291. Inpatient chemotherapy and radiotherapy were estimated to cost £33,379 and £9,878 per year in total, respectively (Table 2).

**Table 2:** Mean total annual costs per category of cost from the payers’ perspective

	Bundled (SD)	Unbundled** (SD)	Chemotherapy (SD)	Radiotherapy (SD)	Total
Inpatient					
Invasive Penile Cancer	£2,314,152 (£85,368)	£34,291 (£18,403)	£33,379 (£18,707)	£9,878 (£11,155)	£2,391,700
Carcinoma in Situ of the Penis	£188,499 (£19,411)	£34 (£76)	£573 (£1,098)	£0 (£0)	£189,106
Outpatient					
Invasive Penile Cancer	£28,571 (£14,689)	£972 (£1,268)	£7,673 (£9,475)	£13,104 (£13,235)	£50,320
Carcinoma in Situ of the Penis	*	*	*	*	*

SD, Standard Deviation

In this table, *corresponds to a figure between 1 and 5. These have been suppressed as per the Hospital Episode Statistics analysis guide

**Excludes chemotherapy and radiotherapy

Outpatients accrued £28,571 bundled costs and £972 for unbundled elements of care. Chemotherapy and radiotherapy delivered in an outpatient setting cost £7,673 and £13,104 per year, respectively.

**Fig. 4:**
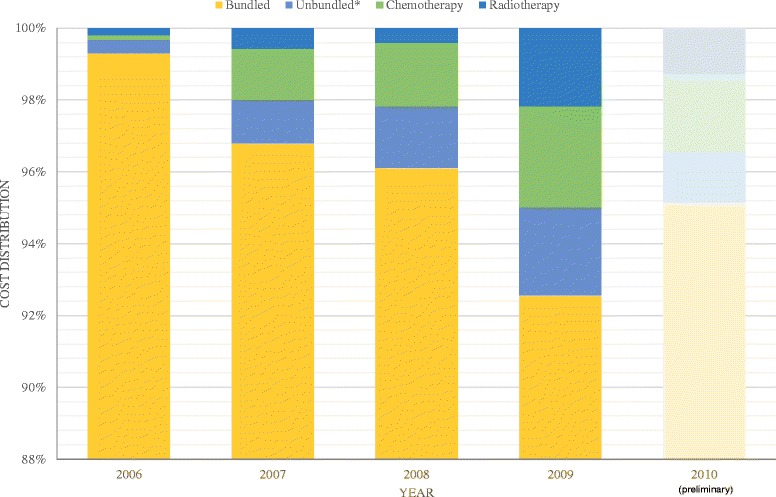
Invasive penile cancer cost distribution per care type. *Excludes chemotherapy and radiotherapy. Note: 2010 figures are based on preliminary data

Bundled costs accounted for 96 % of the total annual burden. Unbundled costs accounted for 1 %, while chemotherapy and radiotherapy accounted for 2 % and 1 %, respectively. This proportion of bundled costs decreased over time from 2006 to 2009 due to improved coding (Fig. 4).

The costs per patient derived from the mathematical model are presented by scenario in Table 3. When using base case inputs in the model (as presented in the tables available in Additional file 1), the cost ranged between £7,421 and £8,063. The lower and upper boundaries, which are based on the uncertainty margins around the base case inputs, are calculated to be between £5,930 and £10,968. In the one way sensitivity analysis, the main contributors to variation in the estimates were found to be the unit costs for interventions and the mode of admission for treatment (Table 4), with costs in all scenarios ranging between £5,531 and £13,050.

**Table 3:** Per patient treatment costs by scenario

	Cost per Patient		
Scenario	Base Case	Lower Bound	Upper Bound
No inflation, no MFF	£7,421	£5,930	£10,104
Inflation, no MFF	£7,465	£5,961	£10,156
No inflation, MFF	£8,015	£6,405	£10,913
Inflation, MFF	£8,063	£6,437	£10,968

MFF, Market Force Factor

**Table 4:** Results of the one way sensitivity analysis by scenario

	Scenarios			
	No inflation, no MFF	Inflation, no MFF	No inflation, MFF	Inflation, MFF
Admission	£5,531-£12,029	£5,573-£12,083	£5,973-£12,991	£6,019-£13,050
Costs^a^	£6,116-£9,746	£6,147-£9,796	£6,606-£10,526	£6,639-£10,580
Intensity of follow-up	£7,133-£7,656	£7,166-£7,704	£7,704-£8,269	£7,739-£8,320
Stage at presentation	£7,304-£7,604	£7,347-£7,650	£7,888-£8,212	£7,935-£8,262
Lymph node management	£7,368-£7,480	£7,412-£7,524	£7,957-£8,078	£8,005-£8,126
Primary tumour management	£7,373-£7,469	£7,418-£7,513	£7,963-£8,067	£8,011-£8,115

MFF, Market Force Factor

^a^Costs for the base case mode of admission were varied

## Discussion

This study provides, to our knowledge, the first estimates of annual and per patient treatment costs for penile cancer in England. Good knowledge of resource use with existing treatments is crucial for the estimation of opportunity costs associated with new treatments. Therefore, this information may be used to inform resource allocation decisions concerning the treatment and prevention of penile cancer in the future.

Our analysis shows that between 2006 and 2010, the mean annual costs of treating invasive penile cancer totalled £3,737 per inpatient and £1,051 per outpatient, with total mean annual costs amounting to £2,442,020 (excluding high cost drugs). The mean cost of a full course of treatment, including follow-up, was estimated to be between £7,421 and £8,063. This latter cost is broadly consistent with what has been observed for other urological cancers, with per patient treatment costs for bladder and prostate cancer estimated to be £8,349 and £7,294, respectively [18].

In the analysis of HES data, information routinely collected in patient medical records from all English hospitals were extracted solely on the presence of penile cancer related ICD-10 codes. Up to twenty diagnosis fields are available per FCE within patient records, thus restricting the presence of an ICD-10 code to the tertiary and secondary diagnosis for inpatients and outpatients may have introduced minor selection bias inherent with case series and furthermore led to potential underestimations. Likewise, costs totally extraneous to penile cancer could have been introduced in the analysis if the diagnosis restrictions were removed.

Furthermore, it is apparent from the results that the HES outpatient dataset is not fully complete, as the vast majority of inpatients would be expected to have at least one single outpatient consultation. Other gaps were found as a result of the system's dependence on clinical coding, especially in the data from earlier years. No costs could be applied to coding errors that arose from such dependence (e.g. missing operational procedure codes, invalid codes for admission or discharge, and absent age fields). Improved training and national audits have augmented this clinical coding process, and hospitals now have explicit incentives for coding data correctly. Nonetheless, these gaps and deficiencies in the dataset have most likely led to some underestimation of the total burden associated with treatment.

There were additional limitations surrounding the use of HRGs and the National Tariff. High cost drugs are excluded and no indication of hospital prescribing data is available within HES and therefore was not considered in this analysis. Moreover, costs associated with unbundled HRGs are not available in the National Tariff due to wide regional variations in resource use and cost. We therefore used the National Reference Costs which provides the foundations for the National Tariff. Definitions for HRGs may vary per year and matching codes and definitions could have led to under- or over-estimations of the economic burden.

The HES data also lacked specificity. It was not possible to determine a patient's stage at diagnosis or track the interventions received through time. Therefore, to estimate total costs per patient we developed a mathematical model to simulate the treatment pathway for the average penile cancer patient. This model was also subject to limitations. Firstly, although local data were applied where possible, the relapse rates used to estimate the transition probabilities in the Markov portion of the model derive from international data, where treatment may not reflect clinical practice in the United Kingdom. The method used to calibrate the model to these data was unable to reflect the uncertainty in these estimates, and any future models would benefit from further exploration of this issue. Furthermore, we were unable to reflect the impact of primary treatment on the likelihood of relapse due to the format of data reporting in the Leijte *et al* study [4].

However, the overall fit of the model to available data was good, with cost estimates broadly consistent with comparable cancer areas. The findings also revealed that costs tended to be insensitive to choices in treatment modality, for example aggressive versus conservative treatment for patients with suspected lymph node involvement, and that the biggest determinant of overall treatment costs is the setting in which care is delivered.

## Conclusion

Despite being rare compared to other malignancies treated by urologists, such as prostate and bladder cancer, penile cancer still has important resource implications which must be accounted for, especially in the context of investment decisions relating to specialist treatment centres. The findings of our study further highlight the potential economic benefits of measures aimed at preventing penile cancers including the promotion of good hygiene practices and HPV vaccination.

## Additional file

Additional file 1:
**Inputs Applied in the Markov Model.** (DOC 299 kb)

## Abbreviations

FCE: Finished Consultant Episode
HES: Hospital Episode Statistics
HPV: Human Papillomavirus
HRG: Healthcare Resource Group
ICD-10: 10th revision of the International Statistical Classification of Diseases and Related Health Problems
