# Supplementary material for: Penile cancer treatment costs in England
Source: BMC Public Health. 2015 Dec 29;15:1305. doi: 10.1186/s12889-015-2669-2 (PMC4696258; doi:10.1186/s12889-015-2669-2)
Supplement: Additional file 1: — Inputs Applied in the Markov Model. (DOC 299 kb) [file 12889_2015_2669_MOESM1_ESM.doc]

**Supplementary Online Material**

Details of the inputs used for the mathematical model are detailed below. Each section includes a brief explanation of the assumptions driving the selection of specific data.

Unless otherwise stated, the average, the lower bound of the 95% confidence interval (CI) and the upper bound of the 95% CI relate to the base case scenario, low cost scenario and high cost scenario, respectively.

1. Referral, Staging and Primary Treatment Probabilities
   1. Referral

Inputs for route of referral were taken from a study of referral patterns into a specialist centre penile cancer centre in the North West of England (Table 1) [1]. All patients were assumed to initially present at a genitourinary medicine clinic or to their general practitioner. Patients visiting genitourinary medicine clinics were assumed to be immediately referred to a urologist, with those initially visiting their general practitioner assumed to either be referred to a urologist or one of the following: a dermatologist, a genitourinary medicine physician or a plastic surgeon. As costs for the latter two specialties were equivalent, patients referred to these were pooled.

Table 1: Source of referral probabilities

| Referral Consultations | *p* | 95% CI | | Reference |
| --- | --- | --- | --- | --- |
| *Initial Consultation* |  |  |  |  |
| Genitourinary physican | 0.061 | 0.056 | 0.065 | [1] |
| *Onward Referral* |  |  |  |  |
| Other specialty | 0.216 | 0.207 | 0.225 | [1] |
| *Other specialties* |  |  |  |  |
| Genito-urinary physican or Plastic surgeon | 0.801 | 0.762 | 0.840 | [1] |

It is likely that some medications would have been prescribed during these consultations with additional costs incurred. However, due to uncertainty in the level of prescribing, only costs for consultations themselves were included in the model.

- 1. Staging and Treatment of Primary Lesion

Post referral patients were all assumed to have an initial face-to-face consultation with a urologist followed by biopsy of the primary lesion, with the results of the latter discussed at a multidisciplinary team (MDT) session.

Table 2: Primary lesion size and grade at referral

| Parameter | *p* | 95% CI | | Reference |
| --- | --- | --- | --- | --- |
| ≧T1G2 | 0.657 | 0.643 | 0.671 | Expert discussion |

Decisions regarding treatment modalities for the primary tumour and likelihood of lymphatic involvement are both related to the size and grade of the lesion, with staging generally carried out using TNM (tumour, node, metastasis) classification from the European Association of Urology Penile Cancer Guidelines [2]. We therefore grouped patients into two categories: those with lesions staged as T1G1 and below (low risk of metastasis/relapse) and those above (intermediate/high risk). Data on the splits between these two groups were provided from North West & North Wales Cancer Network (V Sangar, personal communication) (Table 2).

Primary treatment interventions for the two risk groups are presented in Tables 3 and 4. The proportion of patients undergoing radical treatment (partial or total amputation of the penis) were calculated using data from a 10 year retrospective audit of penile cancer patients treated through the Arden Cancer Network, which covers a population of ~ 1 million [3]. As treatments were only reported by T stage, we assumed that a quarter of amputations carried out on those in the T1 group were for T1G1 tumours, and used this to calculate probabilities and confidence intervals for radical treatment in both risk groups.

Table 3: Primary treatment for low risk patients

| Intervention | *p* | 95% CI | | Reference |
| --- | --- | --- | --- | --- |
| *Treatment type* |  |  |  |  |
| Amputation | 0.095 | 0.061 | 0.129 | [3] |
| *Organ preserving* |  |  |  |  |
| Local excision | 0.697 | - | - | Expert discussion |
| Glansectomy | 0.253 | - | - | Expert discussion |
| Radiotherapy | 0.050 | - | - | Expert discussion |
| *Amputation* |  |  |  |  |
| Total | 0.000 | - | - | Expert discussion |

Organ preserving treatment was assumed to constitute either local excision, glansectomy or radiotherapy. Splits for the different interventions were also obtained from the North West & North Wales Cancer Network (V Sangar, personal communication). All amputations in the low risk group were assumed to be partial, with the ratio in the intermediate/high risk group calculated using the numbers of partial amputations among amputations of all types after removing the assumed number of partial amputations used to calculate the radical treatment proportion in the low risk group from both the numerator and denominator. In the sensitivity analysis, the low cost scenario involved using the upper bound of the 95% confidence interval for organ preserving treatment and partial amputation.

Table 4: Primary treatment for intermediate/high risk patients

| Intervention | *p* | 95% CI | | Reference |
| --- | --- | --- | --- | --- |
| *Treatment type* |  |  |  |  |
| Amputation | 0.665 | 0.612 | 0.718 | [3] |
| *Organ preserving* |  |  |  |  |
| Local excision | 0.697 | - | - | Expert discussion |
| Glansectomy | 0.253 | - | - | Expert discussion |
| Radiotherapy | 0.050 | - | - | Expert discussion |
| *Amputation* |  |  |  |  |
| Total | 0.345 | 0.291 | 0.398 | [3] |

- 1. Staging and Treatment of Lymph Nodes

The two risk groups were further stratified by the clinical stage of the inguinal nodes. In the base case and low cost scenarios, all those in the low risk group were assumed to be cN0, with 5% assumed to have palpable nodes in the high cost scenario. Inputs for the intermediate and high risk group are in Table 5 and were taken from a prospective audit of 100 patients treated per the European Association of Urology guidelines [4].

Table 5: Nodal status in patients with ≥T1G2 tumours

| Parameter | *p* | 95% CI | | Reference |
| --- | --- | --- | --- | --- |
| Palpable nodes | 0.329 | 0.316 | 0.341 | [4] |

Overtreatment of patients with clinically positive lymph nodes is a contentious issue, with some evidence to suggest that a large portion of those who undergo prophylactic lymphadenectomy are unlikely to receive any clinical benefit as a result [4]. Recent changes to treatment guidelines, such as the recommendations around the use of ultrasound guided sentinel node biopsy in intermediate and high risk patients, have attempted to address this problem [2].

The model was designed to reflect current diagnostic, treatment and follow-up approaches for patients based on their lymph node status as accurately as possible. Treatment and follow-up were assumed to be dependent on three interrelated measures: the underlying prevalence of metastatic disease in the relevant populations, and the sensitivity and specificity of the diagnostic tools used to identify those who are likely to benefit from treatment.

Underlying disease prevalence by population can be found in Table 6. For low risk patients with palpable nodes, up to 2% were assumed to have underling the disease after expert discussion. Data for prevalence in patients with T1G2 and above tumours and impalpable nodes was taken from a study evaluating the viability of using ultrasound guided fine needle aspiration to identify nodal disease in patients with penile cancer [5]. Prevalence in patients in the intermediate and high risk group but with palpable nodes was taken from the same study that was used to determine numbers of patients with palpable nodes [4].

Table 6: Underlying prevalence of metastatic disease by risk group

| Parameter | *p* | 95% CI | | Reference |
| --- | --- | --- | --- | --- |
| <T1G2, palpable nodes | 0.010 | 0.000 | 0.020 | Expert discussion |
| ≧T1G2 impalpable nodes | 0.212 | 0.208 | 0.215 | [5] |
| ≧T1G2 palpable nodes | 0.720 | 0.685 | 0.755 | [4] |

The sensitivity and specificity of the different diagnostic tests, by risk group, are presented in Table 7.

Table 7: Sensitivity and specificity of diagnostic tests

| Parameter |  | Sensitivity | Specificity | Reference |
| --- | --- | --- | --- | --- |
| Ultrasound guided fine needle aspiration | | 0.929 | 0.909 | [6] |
| Ultrasound guided sentinel node biopsy | | 1.000 | 0.950 | [2] |

Patients with palpable nodes in either group were assumed to initially undergo ultrasound guided fine needle aspiration (FNA), with proportions of true and false positive and negatives calculated as outlined above. As per the European Association of Urology Guidelines, patients with a negative result were assumed to have a repeat FNA due to the low specificity associated with the procedure. Those with impalpable nodes in the intermediate/high risk group were assumed to undergo sentinel node biopsy.

Positive patients after FNA were assumed to receive additional diagnostic interventions (Computerised Axial Tomography (CT) or Magnetic Resonance Imaging (MRI) scan of the groin and/or chest X-Ray), with probabilities provided by the North West & North Wales Cancer Network (see Table 8). All patients with results which were positive for nodal involvement, whether true or false, were assumed to go on to have either unilateral or bilateral dissection of the inguinal lymph nodes, with a proportion of the latter also undergoing pelvic lymphadenectomy. Probabilities for the different interventions can be found in Table 8.

Table 8: Diagnostic and surgical procedures for patients with lymph node involvement

| Parameter | *p* | 95% CI | | Reference |
| --- | --- | --- | --- | --- |
| Groin Imaging | 0.760 | 0.727 | 0.793 | Expert discussion |
| Of which MRI scans | 0.421 | 0.370 | 0.472 | Expert discussion |
| Chest X-Ray | 0.640 | 0.602 | 0.678 | Expert discussion |
| *Lympdadenectomy* |  |  |  |  |
| Bilateral Inguinal | 0.280 | 0.245 | 0.315 | Expert discussion |
| Pelvic | 0.300 | 0.200 | 0.400 | Expert discussion |

1. Follow-Up
   1. Markov Model Structure

In order to estimate the follow-up treatment costs for penile cancer patients, a Markov model was constructed. The basic structure of the model can be seen in Figure 2 (in main article). All patients were assumed to enter into the disease free state after primary treatment, with monthly probabilities attached to staying in the state, relapsing (local, regional or distant) and dying. Those experiencing relapse were assumed to stay in their respective relapse state for a month, during which time any salvage treatments were assumed to take place, and then either die or move into the associated follow-up state. No excess mortality was assumed for the movement between the relapse and follow-up states. By allowing patients to enter the relapse state in the first month of the model, we were able to account for the fact that some patients were likely to have residual disease after primary treatment despite being classified as disease free for modelling purposes.

The probabilities used in the model were obtained by calibrating its output to published data on relapse and overall expected survival. Calibration targets for relapse were taken from a study by Leijte et al [7]. The target for overall survival was calculated by taking population estimates for five year relative survival [8] and applying them to mortality in the general population [9] over the five year from the mean age of diagnosis (65 years old [8]). Overall model fit was assessed using the sum of the squared residuals and a recursive algorithm was utilised to determine the combination of parameter values, selected from within plausible ranges, which minimised this measure.

Table 9: Parameters of the Weibull distributions covering local and regional relapse

| Parameter |  | *k* | λ | Reference |
| --- | --- | --- | --- | --- |
| Local relapse | | 0.615 | 429.586 | Calibration |
| Regional relapse | | 0.438 | 3393.579 | Calibration |

We initially set out to calibrate the model based on the assumption that all risks of relapse and death were constant over time. However, it quickly became clear that some risks would need to be made time dependent for an acceptable fit to be achieved. After exploring the data further, the Weibull distribution was selected to estimate the monthly probability of local and regional relapse based on the failure times observed in the Leijte et al study, with the latter assumed to fall to zero after five years. All other risks were assumed to be constant, with the risk of distant relapse falling to zero after twenty four months. The final values used in the model as well as the fit achieved can be seen in Tables 9-10 and Figure 3 (in main article).

Table 10: Monthly probabilities used in the Markov model

| Variable |  | Value | Reference |
| --- | --- | --- | --- |
| Disease-free → Distant relapse | | 0.001 | Calibration |
| Disease-free → death | | 0.010 |  |
| Local relapse → post local relapse follow-up | | 0.998 |  |
| Regional relapse → post regional relapse follow-up | | 0.978 |  |
| Distant relapse → post distant relapse follow-up | | 0.869 |  |
| Local relapse → death | | 0.002 |  |
| Regional relapse → death | | 0.022 |  |
| Distant Relapse → death | | 0.131 |  |

- 1. Follow-up interventions
     1. Disease Free Follow-Up

Patients in the low risk group were assumed to not be at risk of any form of relapse, and only all-cause mortality was applied to this group. In the base case, patients in this group were assumed to have an outpatient consultation every four months for the first two years after treatment, six monthly consultations in years three and four and a single consultation in year five. In the low cost scenario, four monthly consultations were assumed to be for the first year only, with six monthly consultation in years two and three and annual consultations in the final two years. In the high cost scenario, patients were assumed to have an outpatient consultation every two months for the first year, every three months in year two, every six months for years three and four and a single consultation in year five.

Patients in the high risk group not experiencing relapse were assumed in the base case to have six consultations in the first two years, four in year three, two in years four and five and annual consultations thereafter, up to ten years. In the low cost scenario, one year of two monthly consultations was followed by two years of three monthly appointments, with two consultations in years four and five. In the high cost scenario, the first three years followed the same patter as the base case except that four consultations were provided in year four.

- - 1. Post-Relapse Follow-Up

All patients experiencing relapse were assumed to be discussed at an MDT meeting before undergoing further treatment. For local relapse, treatment was assumed to be total amputation of the penis, along with a follow-up appointment and ultrasound guided FNA to rule out regional involvement. Although this may slightly overestimate the number of amputations as some patients experiencing local relapse will have already undergone radical surgery, the likely net effect is an underestimation of costs as this small group of patients would likely undergo more costly radiotherapy or chemotherapy. Salvage after regional relapse was assumed to constitute ultrasound guided FNA, followed by lymphadenectomy and adjuvant chemotherapy, with those experiencing distant relapse undergoing palliative treatment only.

Due to the memory-less property of Markov models, it was not possible to model the follow-up costs of those experiencing relapse in the manner used for those remaining disease-free (for all of whom time of entry into the state is known). In order to estimate average monthly follow-up costs for those experiencing local or regional relapse, we took the total costs of follow-up for those in the disease-free state and divided it by the total number of months over which the interventions took place. Monthly costs of follow-up for those with distant relapse were taken as the average of the monthly costs of palliative care observed in a study of a number of different cancer areas [10].

1. Costs

Wherever possible, costs used in the model were taken from the 2010/11 National Tariff [11]. However, as a number of procedures relevant to penile cancer, such as chemotherapy and radiotherapy, are still reimbursed at locally negotiated rates, the 2010/11 References Costs were also used [12].

Two scenarios were examined in the sensitivity analysis. The first involved varying costs without varying the mode of admission, albeit with some exceptions (see below). For costs taken from the Reference Costs, values representing the upper and lower quartiles for specific currency codes were used in the scenarios. Where the National Tariff was used, this approach was not possible, therefore costs were based on adjustments for case mix, for example patients with shorts stays or comorbidities. No adjustments were provided for a number of procedures and as a result, mode of admission was varied for these procedures (biopsies, local excisions and sentinel node biopsies). This scenario was combined with the low and high risk scenarios from the previous sections to provide the range around the base case estimates for overall per patient treatment costs. Details of the costs used in this scenario can be found in Table 11. An additional scenario where the mode of admission was varied was also investigated in the sensitivity analysis. Information on the values used in this scenario can be found in Table 12.

Table 11: Unit costs used in the base, lower and upper cost scenarios

| Consultation/Intervention | Code | Admission | Base Cost | Lower Cost | Upper Cost | Reference |
| --- | --- | --- | --- | --- | --- | --- |
| General practitioner visit | n/a | n/a | £36 | - | - | [13] |
| Initial Genito-Urinary Medicine | 360 | OP | £133 | - | - | [11] |
| Initial Dermatology | 330 | OP | £109 | £88 | £122 | [12] |
| Initial Plastic surgery | 160 | OP | £133 | - | - | [11] |
| Initial Urology | 101 | OP | £194 | - | - | [11] |
| Follow-Up Urology | 101 | OP | £96 | - | - | [11] |
| Initial Clinical Oncology | 800 | OP | £205 | - | - | [11] |
| Follow-Up Clinical Oncology | 800 | OP | £76 | - | - | [11] |
| Initial Medical Oncology | 370 | OP | £282 | - | - | [11] |
| Follow-Up Medical Oncology | 370 | OP | £119 | - | - | [11] |
| MDT | CMDT_LG | OP | £152 | £37 | £160 | [12] |
| Biopsy | LB32B/A | CDCE/NE | £706 | £526 | £884 | [11] |
| Fine needle Aspiration | WA24Z | OP | £211 | £137 | £292 | [12] |
| Sentinel Node Biopsy | QZ18Z | CDCE/NE | £1,733 | £1,733 | £4,155 | [11] |
| CT scan | RA12Z | OP | £133 | £103 | £153 | [12] |
| Ultrasound | RA23Z | OP | £53 | £39 | £61 | [12] |
| MRI Scan | RA05Z | OP | £319 | £182 | £410 | [12] |
| Local excision | LB32B/A | CDCE/NE | £706 | £526 | £884 | [11] |
| Glansectomy | LB31Z | CDCE | £2,263 | £2,263 | £2,689 | [11] |
| Radiotherapy Planning | SC52Z | DC | £1,309 | £674 | £1,309 | [12] |
| Radiotherapy Delivery | SC23Z | DC | £134 | £122 | £134 | [12] |
| (number of fractions) |  |  | 15 | 10 | 20 | Expert discussion |
| Chemotherapy procurement | SB05Z | DC | £614 | £251 | £803 | [12] |
| Chemotherapy initial delivery | SB14Z | DC | £334 | £232 | £401 | [12] |
| Chemotherapy subsequent delivery | SB15Z | DC | £294 | £210 | £343 | [12] |
| (number subsequent sessions) |  |  | 2 | 1 | 2 | Expert discussion |
| Partial Amputation | LB31Z | CDCE | £2,263 | £2,263 | £2,689 | [11] |
| Total Amputation | LB31Z | CDCE | £2,263 | £2,263 | £2,689 | [11] |
| Block Dissection of Inguinal Lymph Nodes | FZ12B/A/C | CDCE | £2,755 | £1,970 | £3,374 | [11] |
| Block Dissection of Pelvic Lymph Nodes | FZ12B/A/C | CDCE | £2,755 | £1,970 | £3,374 | [11] |

OP, outpatient; CDNE, combined day case elective; NE, non-elective; DC, day case.

Table 12: Values used in scenario where mode of admission was varied

| Consultation/Intervention | Low Cost Admission | | | Low Cost Admission | | | Reference |
| --- | --- | --- | --- | --- | --- | --- | --- |
|  | Code | Admission | Base Cost | Code | Admission | Base Cost |  |
| MDT | CMDT_SpG | OP | £145 | CMDT_C | OP | £165 | [12] |
| Biopsy | OP | CZ36Y | £177 | LB32A | NE | £2,104 | [11], [12] |
| Fine needle Aspiration | WA24Z | OP | £0 | WA24Z | OP | £422 | [12] |
| Sentinel Node Biopsy | LB32B | CDCE | £706 | LB31Z | CDCE | £2,263 | [11] |
| CT scan | RA10Z | OP | £100 | RA13Z | OP | £151 | [12] |
| Ultrasound | RA23Z | OP | £0 | RA23Z | OP | £106 | [12] |
| MRI Scan | RA037 | OP | £218 | RA07Z | OP | £357 | [12] |
| Local excision | CZ36Y | OP | £177 | LB32A | NE | £2,104 | [11], [12] |
| Glansectomy | LB31Z | CDCE | £2,263 | LB48Z | E | £2,934 | [11], [12] |
| Radiotherapy Planning | SC52Z | OP | £729 | SC52Z | E | £2,869 | [12] |
| Radiotherapy Delivery | SC23Z | OP | £111 | SC23Z | E | £261 | [12] |
| Chemotherapy procurement | SB05Z | OP | £607 | SB05Z | E | £579 | [12] |
| Chemotherapy initial delivery | SB14Z | OP | £302 | QZ18Z | CDCE | £1,733 | [11], [12] |
| Chemotherapy subsequent delivery | SB15Z | OP | £206 | QZ18Z | CDCE | £1,733 | [11], [12] |
| Partial Amputation | LB31Z | CDCE | £2,263 | LB47Z | E | £4,320 | [11], [12] |
| Total Amputation | LB31Z | CDCE | £2,263 | LB47Z | E | £4,320 | [11], [12] |
| Block Dissection of Inguinal Lymph Nodes | QZ18Z | CDCE | £1,733 | FZ12B | NE | £4,051 | [11] |
| Block Dissection of Pelvic Lymph Nodes | QZ18Z | CDCE | £1,733 | FZ12B | NE | £4,051 | [11] |

OP, outpatient; CDNE, combined day case elective; NE, non-elective; E, elective.

**References**

[1] Lucky MA, Rogers B, Parr NJ. Referrals into a dedicated British penile cancer centre and sources of possible delay. Sex Transm Infect 2009;85:527–30.

[2] Pizzocaro G, Algaba F, Horenblas S, Solsona E, Tana S, Van Der Poel H, et al. EAU penile cancer guidelines 2009. Eur Urol 2010;57:1002–12.

[3] Mistry T, Jones RWA, Dannatt E, Prasad KK, Stockdale AD. A 10-year retrospective audit of penile cancer management in the UK. BJU Int 2007;100:1277–81.

[4] Hegarty PK, Kayes O, Freeman A, Christopher N, Ralph DJ, Minhas S. A prospective study of 100 cases of penile cancer managed according to European Association of Urology guidelines. BJU Int 2006;98:526–31.

[5] Ivaz S, Lam W, Swallow T, Corbishley C, Perry M, Pilcher J. A prospective study to evaluate the performance of ultrasound with or without fine needle aspiration of inguinal nodes in patients with squamous cell carcinoma of the penis. J Urol 2010;183:e224.

[6] Saisorn I, Lawrentschuk N, Leewansangtong S, Bolton DM. Fine-needle aspiration cytology predicts inguinal lymph node metastasis without antibiotic pretreatment in penile carcinoma. BJU Int 2006;97:1225–8.

[7] Leijte JAP, Kirrander P, Antonini N, Windahl T, Horenblas S. Recurrence patterns of squamous cell carcinoma of the penis: recommendations for follow-up based on a two-centre analysis of 700 patients. Eur Urol 2008;54:161–8.

[8] Verhoeven RHA, Janssen-Heijnen MLG, Saum KU, Zanetti R, Caldarella A, Holleczek B, et al. Population-based survival of penile cancer patients in Europe and the United States of America: no improvement since 1990. Eur J Cancer 2013;49:1414–21.

[9] Office for National Statistics. England and Wales Interim Life Table, 1980-82 to 2008-10 2010.

[10] Guest JF, Ruiz FJ, Greener MJ, Trotman IF. Palliative care treatment patterns and associated costs of healthcare resource use for specific advanced cancer patients in the UK. Eur J Cancer Care (Engl) 2006;15:65–73.

[11] Department of Health. 2010-11 National Tariff 2011.

[12] Department of Health. 2010-11 Reference Costs 2011.

[13] Curtis L. Unit Costs of Health and Social Care. PSSRU: Kent; 2011.
